# Reimagining Kangaroo Care for Preterm Infants: A Novel Garment for Safe and Comfortable Bonding

**DOI:** 10.3390/children11111392

**Published:** 2024-11-16

**Authors:** Alexandra McMillin, Aviva Presser Aiden, Jules P. Sherman, Ruth Ann Crystal, William D. Rhine

**Affiliations:** 1 Lucile Packard Children’s Hospital Stanford, 453 Quarry Road, Palo Alto, CA 94304, USA; amcmil17@uw.edu (A.M.); axaiden@texaschildrens.org (A.P.A.); rcrystal@stanford.edu (R.A.C.); wrhine@stanford.edu (W.D.R.); 2 Children’s National Hospital, 111 Michigan Avenue NW, Washington, DC 20010, USA

**Keywords:** kangaroo care, skin-to-skin care, NICU, preterm baby care, neonatology, neonatal care, line management, pediatric care, acute care settings, premature infant care, product design, innovation, pediatric medical device

## Abstract

Background/Objectives: Kangaroo Care (KC) has been proven to enhance physiological stability, growth, and bonding in preterm, low-birthweight infants. Despite its benefits, KC is underutilized in Level IV Neonatal Intensive Care Units (NICUs) due to challenges in managing medical equipment. This study introduces the Kangarobe™, a novel garment designed to facilitate safe, comfortable, and efficient KC for medically fragile infants in high-acuity NICUs. Methods: From 2021 to 2023, a feasibility study was conducted involving 25 infant-parent dyads in a Level IV NICU. The Kangarobe™ was designed using human-centered design principles and tested on infants dependent on respiratory support. Surveys employing a 5-point Likert scale were administered to parents and nursing staff to assess safety, comfort, ease of use, and procedural access. Results: Survey results showed positive feedback from both parents and nursing staff, particularly in the areas of safety and comfort. For example, 72–80% of parents and nurses responded positively regarding ease and comfort. High level of agreement (76%) on the security of medical line management, with minimal negative feedback. In addition, parents using the Kangarobe™ held their infants for an average of 171 min per session, with a notable increase compared to the typical 75 min, indicating enhanced comfort and feasibility for extended KC sessions. The Kangarobe™ successfully enabled the secure management of medical lines and tubes, with the vertical access window improving procedural efficiency without interrupting KC. Conclusions: The Kangarobe™ demonstrates promise in addressing barriers to KC in high-acuity NICUs. By enhancing safety, comfort, and ease of use, it supports wider adoption of KC practices, potentially improving patient safety, staff efficiency, and family-centered care.

## 1. Introduction

Kangaroo Care (KC), a method of skin-to-skin contact between parent and infant, has gained considerable attention for its profound benefits on preterm infants in the NICU. With over four decades of empirical evidence, KC has proven effective in enhancing parent-infant bonding, promoting weight gain, stabilizing respiratory and cardiac functions, supporting neurological development, and reducing stress levels in both mother and infant [1,2,3,4]. A recent review of 31 trials with 15,559 infants showed that compared to conventional care, KC reduces mortality risks during birth hospitalization and decreases rates of severe infection. The decrease in infant mortality with KC was observed regardless of gestational age or enrollment weight [5]. However, the application of KC is limited for infants who require complex medical equipment, such as intravenous lines, nasogastric tubes, and respiratory support. Neonatal Intensive Care Units (NICUs) are categorized into different levels based on the complexity of care they provide, with Level IV being the highest. Level III and IV NICUs offer the most advanced care for premature and sick newborns, equipped to handle the most complex and high-risk situations with state-of-the-art technology and an interdisciplinary team. In the neonatal intensive care unit (NICU), nurses play a crucial role in providing support to parents, especially concerning Kangaroo Care (KC). This skin-to-skin practice is not only critical for the infant’s physiological stabilization and growth but also for nurturing the maternal bond which is crucial in a high-tech, high-intensity care environment [6,7]. Recent literature underscores the urgent need for ergonomically designed, safe support products tailored for the unique challenges of the high acuity (Level III and Level IV) NICU environment [8]. These products should aim to facilitate a comfortable experience for both the parent and the critically ill infant, while securely managing multiple medical lines without compromising parental comfort. At present, many NICUs resort to makeshift solutions like taping lines and tubes to parents or chairs, coupled with blanket coverage for warmth. These ad hoc methods raise significant safety concerns, including the risk of accidental falls, medical equipment dislodgement, and unplanned extubation. Moreover, the lack of standardization exacerbates the inconsistency in securing medical devices effectively.

Additional challenges in the widespread adoption of KC include limited awareness of its benefits, time constraints, staff and parent anxiety, inadequate training, and a dearth of specialized KC support devices [9,10]. Products currently on the market include The Zaky Zak^®^ Joeyband™ and The Moby Wrap^®^. These examples resemble tube tops or baby-carrying wraps which may help facilitate KC with babies; however, they are not specifically designed for high-acuity infants with multiple lines and tubes that need to be managed while performing KC. Clinician surveys have identified key design features essential for a successful KC device, such as secure infant holding mechanisms, washability, and easy access to medical procedures [11].

To address these multifaceted needs, we crafted survey questions based on barriers faced by both clinicians and parents while performing KC in a high acuity setting and employed design thinking methodology, incorporating invaluable insights from NICU clinicians and parents, to develop the Kangarobe™. 

This feasibility study aimed to evaluate the utility and potential benefits of the Kangarobe™ in high acuity settings with babies who have multiple lines and tubes, in comparison to standard practice, as described above. Specifically, the study focuses on evaluating the duration of transfer for KC, ease of use, comfort levels for the parent-infant dyad, security of support devices, and procedural accessibility. 

## 2. Methods

The inventors applied Design Thinking (DT), also known as ‘human-centered design’, in developing the Kangarobe™. (Table 1). This approach prioritizes addressing unmet human needs as the primary objective, with its framework specifically structured to achieve this goal [12,13]. The design-thinking framework progresses through three main stages: (1) understanding, (2) exploring, and (3) materializing. These stages encompass six phases: empathize, define, ideate, prototype, test, and implement [13].

### 2.1. Device Features and Materials

**Figure 1:**
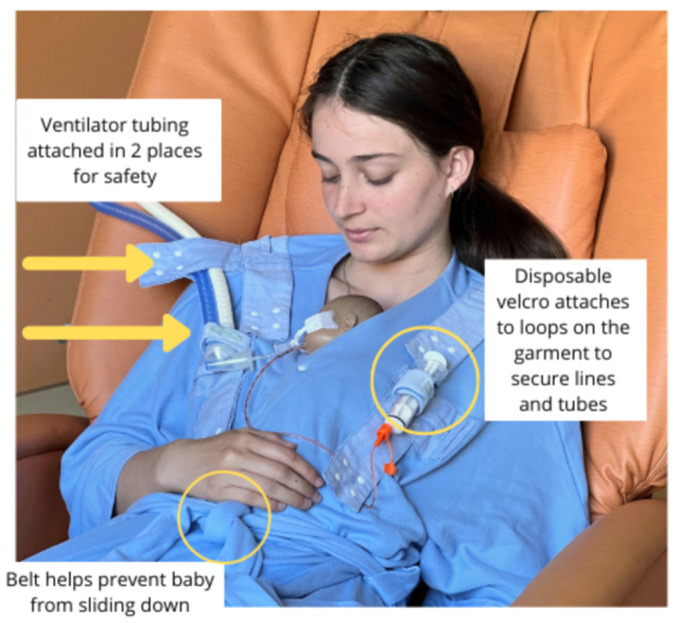
Simulation of KC using the Kangarobe™ for an intubated manikin baby connected to ventilator tubing. The Kangarobe™ garment includes snap fasteners and loops that attach to disposable Velcro^®^ points of attachment to secure ventilator tubing, feeding tubes, and intravenous lines, a belt used for support under the baby, and snaps at the neckline to keep parent and baby covered.

The Kangarobe™ is a soft, full-coverage garment that functions akin to a robe, designed for parents to safely engage in KC with their infant in the NICU [Figure 1]. Crafted from a machine-washable blend of 60% cotton and 40% polyester, the garment offers a versatile one-size-fits-most design. For enhanced positional support, a strategically placed belt can be placed beneath the baby. Engineered for adaptability, the Kangarobe™ features an array of snap fasteners and loops strategically located across the shoulders and chest. Loops are used with disposable foam wraps or bracelets with single-use Velcro^®^, allowing for the secure placement of several types of respiratory, feeding, and vascular tubing. The garment is designed to support various respiratory devices, including low and high-flow nasal cannulas, continuous positive airway pressure (CPAP) systems, and ventilator tubing. Opting for disposable Velcro^®^ closures over permanent ones mitigates the risk of unsanitary debris accumulation in the permanent Velcro^®^, thereby ensuring long-term effectiveness.

**Figure 2:**
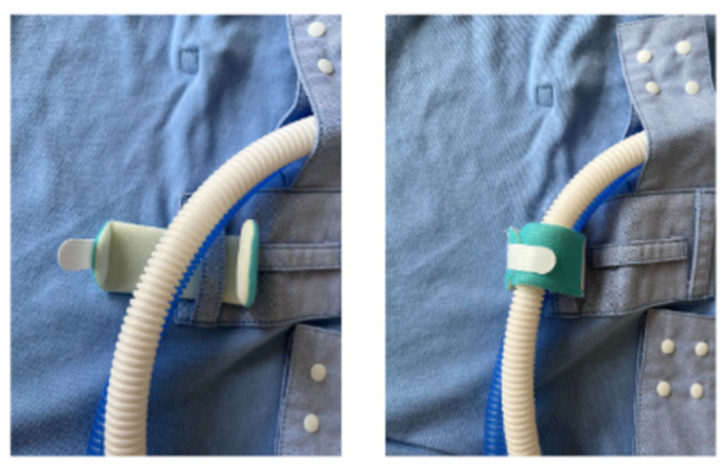
Close-up view of the disposable Velcro^®^ securing respiratory tubing.

**Figure 3:**
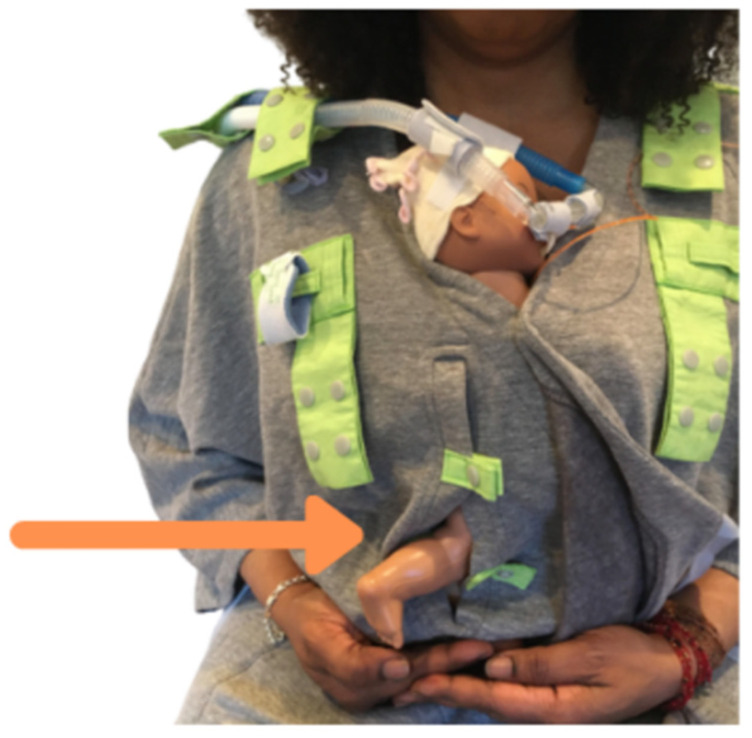
The vertical access window allows nurses to do heel sticks or checks on the baby without disturbing KC and while keeping the parent covered.

Among its patented innovations, the Kangarobe™ incorporates multiple snaps and attachment loops [Figure 2], as well as a vertical access window [Figure 3]. This unique feature grants clinicians unobtrusive access to the infant for medical evaluations and interventions, all while maintaining the integrity of the KC experience. Additionally, adjustable snaps on the neckline ensure that parents remain covered during medical procedures on the baby, further enhancing the garment’s utility and comfort.

### 2.2. User Survey Methods

The Kangarobe™ garment was evaluated with 25 parent-infant pairs from 1 September 2021 to 31 August 2023, in the level IV NICU at Lucile Packard Children’s Hospital Stanford. The study received approval from the Stanford University Institutional Review Board, protocol ID 60251. Participants were eligible if the parent had experience holding skin-to-skin at least three times and the infant was dependent on a respiratory support device. Informed consent was obtained from study participants. Parents and nurses were provided instructions by a research assistant on how to put on and use a freshly laundered Kangarobe™. Additionally, both parents and nurses were provided access to an instructional video available in both English and Spanish. The data collected included the start and end times of KC, and the number of staff engaged in set up and return of infants. For the last seven patients, temperature prior to and after KC was recorded. 

**Figure 4:**
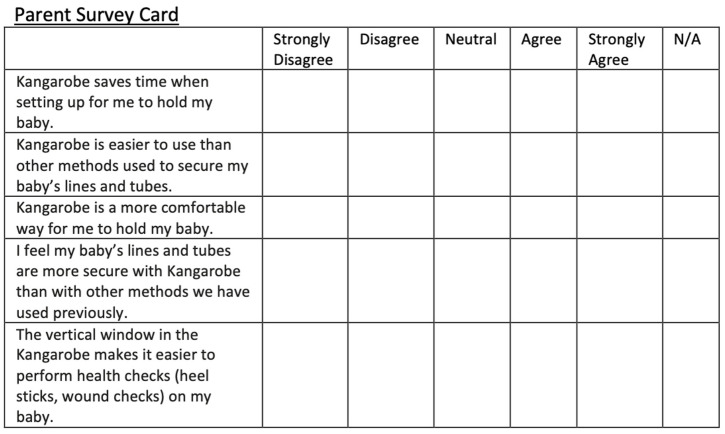

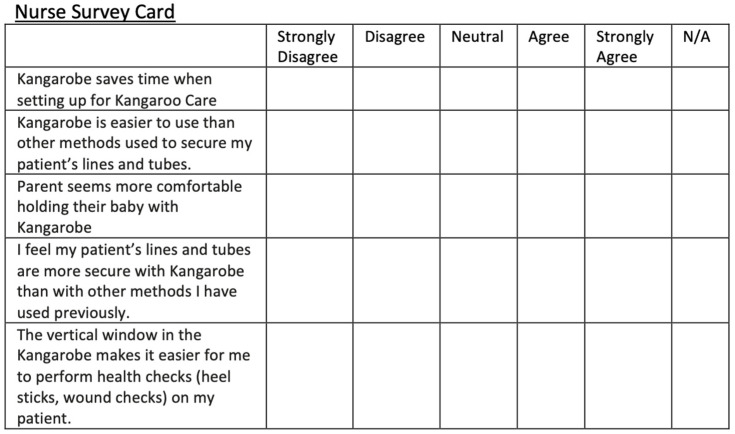
Parent and Nurse Surveys using a 5-point Likert Scale.

We developed a qualitative survey for parents and nurses ensuring the questions would capture the experiences, perceptions, and insights of the participants. Questions were developed by defining our objectives (understanding ease of use, comfort, and safety for babies connected to multiple lines and tubes) and pilot testing them to be sure they were clear and provided us with valuable data. After KC using the Kangarobe™ (Gold Health, LLC, Palo Alto, CA, USA), both parents and nurses were surveyed using a 5-point Likert scale to collect responses regarding time efficiency, safety, comfort, ease of set up/use, and procedure access. Participants could respond with the following: “strongly agree”, “agree”, “neutral”, “disagree”, or “strongly disagree”. Nurses and parents were given similar surveys. (Figure 4) Parents and nurses were also given the opportunity to provide verbal or written feedback about their experiences with the Kangarobe™ (Gold Health, LLC, Palo Alto, CA, USA).

## 3. Results

### 3.1. Design Feedback Results

Comments in Table 2 and Table 3 were integral to the iterative design process for the Kangarobe. This feedback was given throughout our design process.

### 3.2. User Survey Results

The pilot study of the Kangarobe™ included a convenience sample of 23 mother-baby, and 2 fathers-baby dyads, and reflected our NICU demographics with multiple ethnicities and approximately 50% with public insurance. In this human pilot study, the infants had an average corrected gestational age of 33 weeks (ranging from 25 to 42 weeks) and a mean weight of 1.145 kg (ranging from 0.64 kg to 3.56 kg). Out of 25 infants in the study, 19 were on CPAP and 6 were on HFNC. The Kangarobe™ garment is compatible for use with intubated babies, but no intubated participants were recruited in this study. Of the total participants, all babies had gastric tubes, and 6 had IV lines, including three PICC and three peripheral intravenous (PIV) lines.

Transferring infants to parents wearing the Kangarobe™ and back to their bed took an average of 4.2 min and 4 min, respectively. Parents held using the Kangarobe™ for 171 min per instance on average, with 308 min being the longest hold and 20 min being the shortest hold. For comparison, based on quality improvement data, the median duration of KC by parents in the Stanford NICU in 2022 was 75 min per instance. There were no safety events reported in this study with the use of the Kangarobe™. For the last seven participants, the baby’s axillary temperature was taken prior to and after KC. The temperature difference on average was an increase of 0.1C, excluding one outlier having a 2.3 °C temperature decrease (from 38.6 prior to 36.3 after).

**Figure 5:**
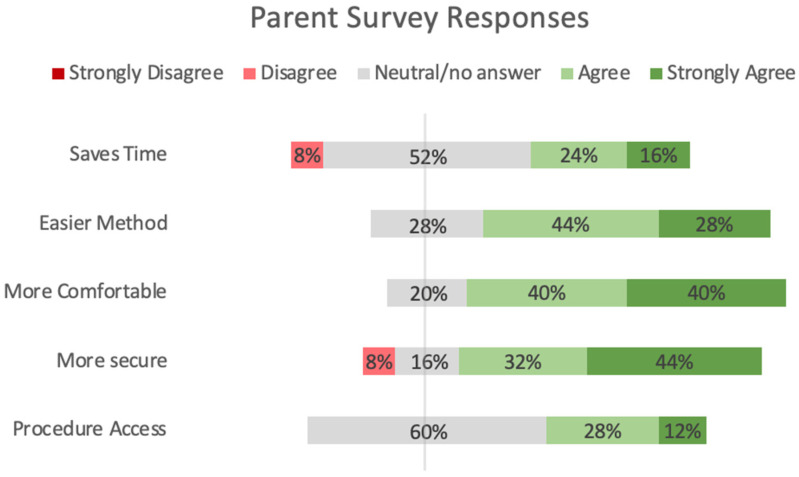
Parent Survey Responses.

Figure 5 shows the results from parents and surveys comparing the Kangarobe™ to the traditional ad hoc method of KC. Survey questions were categorized into five domains: time savings, ease of use, comfort, security, and procedure access (via vertical access window). There was a preponderance of positive responses, “strongly agree” or “agree” (72% and 80%, respectively), and no negative responses in the domains of ease of use and comfort. For the security of lines, there was still a predominantly positive response (76%), with four (16%) neutral responses and two (8%) negative (“disagree”) responses. Twelve parents did not answer the question about procedure access, either because it was not used or because that question was deferred to the nursing staff. Of those 13 parents who responded, 40% were positive, 60% were neutral, and none were negative.

**Figure 6:**
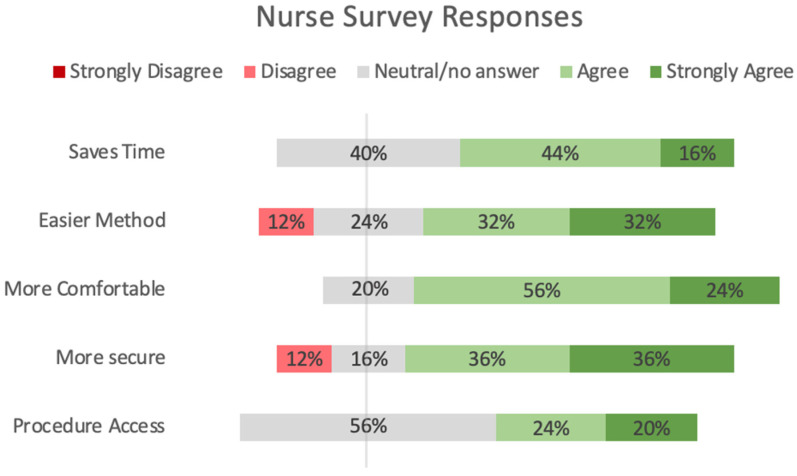
Nurse survey responses.

Similarly, Figure 6 shows the results from the nurse surveys in the same five domains. For them, there were predominantly positive responses for time savings and comfort, 60% and 80%, respectively, with the remainder being neutral. For ease of use and security, positive responses dominated (64% and 72%), with some neutral responses (24% and 16%) and a few (12%) negative (“disagree”) answers. Not all nurses answered the question regarding the procedure access via the vertical window; of the 16 who did, 44% were positive, 56% were neutral, and none were negative.

The chart illustrates a summary of the 25 nurse responses to the survey. Neutral responses to question 5 about the vertical access window may reflect that it was not used. No one who used the vertical access window disliked it.

## 4. Discussion

Extensive research has demonstrated that KC in the NICU is closely associated with improved health outcomes for premature infants, both in the short and long term [14]. Despite its many benefits, KC for premature infants is underutilized due to several barriers including concerns regarding safety, comfort, and privacy during transfer and holding, and security of baby’s lines and respiratory tubing.

Input from NICU staff and parents was integral to the iterative design process for the Kangarobe™. Gathering user feedback early and frequently throughout the rapid prototyping process is crucial to identify promising solutions and refine prototypes quickly for further testing. While this feasibility study is limited in size and scope, we have illustrated that the Kangarobe™ is promising as a safe and efficient KC device for babies with multiple lines and tubes. The results suggest that the implementation of the Kangarobe™ offers an improved KC experience for infants, nurses, and parents in the NICU, with the potential for improving patient safety, healthcare staff efficiency, and supporting family-centered care.

The involvement of nurses is crucial, as they are integral in guiding and supporting mothers through the KC process. Nurses provide emotional support and practical assistance, teaching mothers how to provide primary care such as breastfeeding and hygiene, and specialized care such as oral drug administration. Further, KC initiated early and practiced often provides numerous benefits. Mothers report enhanced attachment, increased milk production, and more successful breastfeeding, while fathers also report increased bonding and confidence in providing care. Notably, both parents experience decreased levels of anxiety during KC, and infants exhibit decreased stress responses [7].

In reviewing the survey data, several key insights emerge that warrant discussion. Despite the limited participant pool, certain trends and feedback patterns offer valuable preliminary insights. Notably, participants consistently reported positive responses to the intervention’s usability and comfort, reinforcing existing literature on its acceptability and potential for enhancing infant care. However, some feedback highlighted specific limitations, such as occasional discomfort during extended use, suggesting areas for refinement in future designs. These insights, though based on a small cohort, align with the known benefits of similar interventions, adding to the body of evidence supporting its use in neonatal care. The survey results also indicate directions for future research, such as further testing with a larger, more diverse population to validate these initial findings and explore demographic variations. Additionally, this feedback provides a basis for practical adjustments, ensuring the intervention meets the needs of caregivers and healthcare providers more effectively.

The Kangarobe™ has the potential to significantly impact clinical workflow and nursing workload in the NICU. By providing a garment designed for safe and continuous skin-to-skin contact, it could streamline care procedures by reducing the need for constant repositioning and monitoring typically required for preterm infants. This enhanced stability could allow nurses to feel more confident leaving infants in the Kangarobe™ for longer durations, thereby freeing up time for other critical tasks. Additionally, the Kangarobe™‘s compatibility with various respiratory devices, such as nasal cannulas and ventilator tubing, could simplify device management, reducing the complexity and frequency of adjusting equipment during skin-to-skin sessions. This efficiency may not only enhance the quality of care provided to each infant but could also lower the physical and cognitive demands on nurses, potentially reducing fatigue and improving overall workflow in the NICU. Moreover, by providing a safer and more effective means for parents to engage in Kangaroo Care, the Kangarobe™ supports family involvement in the caregiving process, potentially lightening the emotional workload on nursing staff by sharing some aspects of care with parents. However, further research is needed to quantify these potential benefits and assess the Kangarobe™’s impact on NICU workflow and nursing efficiency.

The integration of the Kangarobe™ can enhance the KC experience for infants, nurses, and parents in the NICU. By addressing the logistical challenges associated with traditional KC, the Kangarobe™ can facilitate increased adoption of this beneficial practice. Notably, this innovation also holds the potential to enhance patient safety, and healthcare staff efficiency, and support family-centered care, provided that nurses continue to play their supportive role efficiently and compassionately in the NICU [8].

## 5. Limitations

There are limitations to this study that need to be acknowledged. The participant pool in this study was limited, and a larger cohort would be advantageous to validate these findings. Additionally, there needs to be a demonstration of safe use with higher acuity support devices (endotracheal tubes and tracheostomy tubes) whose stability is more critical to patient safety compared to lower acuity devices. With simulation training sessions nurses may be more comfortable and confident with the use of the Kangarobe™. Furthermore, some parents were significantly more comfortable with KC due to having more extensive experience transferring the infants from bed to parent. This could impact the data by lowering the transfer time. Finally, we did not compare the duration of the hold of each individual parent performing KC with the Kangarobe™ versus using a different garment with subsequent holding events.

## 6. Conclusions

Kangaroo Care (KC) has significant benefits for preterm and infants with low birth weight admitted to the NICU. To facilitate the widespread use of KC in the NICU, it is important for the NICU to have readily available support devices, which facilitate a comfortable experience for the parent and infant, while also securing lines that support the baby. In this study, we tested the Kangarobe™, a belted wrap garment designed to facilitate safe and efficient caregiver skin-to-skin holding for babies in the NICU with multiple medical devices. The garment integrates multiple snap fasteners and loops with disposable Velcro^®^ to secure medical support devices the infant requires, as well as an easy-access vertical window to allow evaluations of the infant including heel sticks and wound checks without disturbing KC. The Kangarobe™ provides multiple secure attachment points for medical lines and ventilation tube routing, safe infant support while the parent holds, and easy access to the baby by nurses. The highly positive user responses from both NICU nurses and parents demonstrate the benefits of using their feedback to design a garment to better support KC.

## Figures and Tables

**Table 1:** Phases of the Design Thinking Model.

Phase	Description
EMPATHY/ UNDERSTAND	The first phase of the design thinking process involves developing a thorough understanding, often referred to as ‘contextual inquiry’. This phase immerses investigators in learning through methods such as interviews, focus groups, and observations of people and processes. The objective is to step into the users’ shoes, fostering empathy and a comprehensive grasp of the problem to be solved.
DEFINE	During the ‘define’ phase, the emphasis is on identifying the unmet needs experienced by users. Problem statements often take the form of ‘How might we…?’ questions, capturing insights derived from the contextual inquiry.
IDEATE	Ideation, or brainstorming, is an essential part of design thinking, focusing on creativity and collaboration. During this phase, it is important to withhold judgment and promote ‘out-of-the-box’ ideas. A useful technique for fostering open communication is to start responses with ‘Yes, and...’. instead of ‘No, but...’.
PROTOTYPE	A prototype is an inexpensive, low-fidelity model of a proposed solution designed to convey ideas quickly. It can take various forms, such as a sketch, diagram, model, 3D-printed object, skit, or intangible intervention. Prototypes are tested in rapid cycles with small groups to gather feedback and make iterative improvements. This agile testing approach helps the team identify feasible solutions that can be further explored on a larger scale.
TEST	Testing is an iterative process involving real users, offering the team valuable feedback. The goal is to identify what works and what does not, allowing for rapid refinement.
IMPLEMENT	The refined solution into practice is put into practice. This phase allows designers to understand how their innovation integrates (or does not) into a current workflow with multiple stakeholders, and the healthcare system at large.

**Table 2:** Nurse Feedback.

Nurse Feedback
“It would be nice to have better coverage for moms. Maybe more like a robe you see in a spa. Most of our moms feel uncomfortable being in a tube top in the middle of the NICU, they don’t want to do KC with their babies that way”.
“Velcro^®^ used in bunting is a pain because it collects all the fibers and debris. I like that you did not use Velcro^®^”.
“I check a multitude of things hourly on the baby including the peripherally inserted central catheter (PICC) line, epidural, if the baby has one, wounds and drains. I like the idea of opening [in the fabric] to peek in to see the baby without unswaddling them”.
“I like the vertical window. Kangaroo care decreases pain with procedures. It will be easier to do heel sticks with Mom holding”.
“I like the idea of a way to check on the baby without interrupting KC, but it was harder to check the baby’s feet with the window being horizontal. Making the window up and down [vertical] would allow us to check a wound or epidural or do heel sticks on the baby more easily”.
“This [Kangarobe system] is much more secure [than standard practice]. The baby was much less fussy with the Kangarobe™”.
“This [the Kangarobe™] is much better than stretchy shirts. The Kangarobe™ was more comfortable for the mom and the baby”.
“Clips are one more thing to lose. If something is sewn onto the gown, there is more chance that the nursing staff will comply”.
“If tape residue gets on the fabric, we have to throw that gown away”.
“You should put tags on the edge of the snaps [on the vertical access window] to help nurses open them with their gloves on”.

**Table 3:** Mother Feedback.

Mother Feedback
“This [the Kangarobe™] is much better than taping the tubes to the chair. Before the nurses struggled for 15 min looking for where to put the different things (lines and tubes). This time transfer of the baby (using the Kangarobe™) was a lot faster and only took a few minutes. The baby was crying for less time and settled down more quickly”.
“Before, I was given a gown to put on backwards so that it opened in the front for KC. There were no buttons or snaps and I felt very exposed. I feel a lot less exposed in the Kangarobe, which I like”.
“When my baby had total parenteral nutrition (TPN), the nurses had me hold the central line in my hand for kangaroo care. That was scary. I did not want to do that”.
“When they taped the tubing to the chair, my baby was uncomfortable because as he moves, his CPAP moves too, and it pulls on his nostrils. Subsequently, she [the nurse] had to readjust the nasal prongs”.
“I could see that he (baby) looked more comfortable [with the Kangarobe™]. Usually, the CPAP nasal prongs push on his mouth, and I have to adjust them periodically, but this time the CPAP was stable.
“I noticed that there was less condensation in the CPAP tubing. Usually, we have to empty the CPAP pooling with water from the tubing, 3 to 4 times per KC session, but this time we only had to empty it 1 time, which was better” [Ventilation tubing was attached to one shoulder of the Kangarobe™, looped behind the mom’s neck and attached in a second place on the mom’s other shoulder.]
“It would be great if there were a slit [in the fabric] to be able to pump milk while holding my baby. My milk production often increases when I hold my baby”.
“The Kangarobe™ was very comfortable. The biggest difference was with the CPAP. The Kangarobe held the CPAP tubing in place better and my baby could relax and sleep better”.
“Also, I had a C-section [cesarean section delivery] and I like that the Kangarobe™ does not push on my incision”.

## Data Availability

The data supporting the findings of this study are not publicly available due to patient privacy concerns governed by HIPAA regulations.

## Patent

U.S. Patent No. 11,819,142 · Issued 21 November 2023.

## References

[1] Ruiz-Peláez J.G., Charpak N., Cuervo L.G. (2004). Kangaroo Mother Care, an example to follow from developing countries. BMJ.

[2] Cristóbal Cañadas D., Parrón Carreño T., Sánchez Borja C., Bonillo Perales A. (2022). Benefits of Kangaroo Mother Care on the Physiological Stress Parameters of Preterm Infants and Mothers in Neonatal Intensive Care. Int. J. Environ. Res. Public Health.

[3] Charpak N., Ruiz J.G., Zupan J., Cattaneo A., Figueroa Z., Tessier R., Cristo M., Anderson G., Ludington S., Mendoza S. (2005). Kangaroo Mother Care: 25 years after. Acta Paediatr..

[4] Ludington-Hoe S.M. (2011). Evidence-based review of physiologic effects of kangaroo care. Curr. Women’s Health Rev..

[5] Sivanandan S., Sankar M.J. (2023). Kangaroo mother care for preterm or low birth weight infants: A systematic review and meta-analysis. BMJ Glob. Health.

[6] Eskandari S., Mirhaghjou S.N., Maryam Maleki M., Mardani Gholami M., Harding C. (2021). Identification of the Range of Nursing Skills Used to Provide Social Support for Mothers of Preterm Infants in Neonatal Intensive Care. Crit. Care Res. Pract..

[7] Maleki M., Mardani A., Harding C., Basirinezhad M.H., Vaismoradi M. (2022). Nurses’ strategies to provide emotional and practical support to the mothers of preterm infants in the neonatal intensive care unit: A systematic review and meta-analysis. Women’s Health.

[8] Saptaputra S.K., Kurniawidjaja M., Susilowati I.H., Pratomo H. (2021). How to improve the effectiveness and efficiency of Kangaroo Mother Care: A literature review of equipment supporting continuous Kangaroo Mother Care. Gac. Sanit..

[9] Penn S. (2015). Overcoming the barriers to using kangaroo care in neonatal settings. Nurs. Child. Young People.

[10] Seidman G., Unnikrishnan S., Kenny E., Myslinski S., Cairns-Smith S., Mulligan B., Engmann C. (2015). Barriers and enablers of kangaroo mother care practice: A systematic review. PLoS ONE.

[11] Weber A., Jackson Y. (2021). A Survey of Neonatal Clinicians’ Use, Needs, and Preferences for Kangaroo Care Devices. Adv. Neonatal Care.

[12] Gaulton J., Crowe B., Sherman J. (2023). How Design Thinking and Quality Improvement Can Be Integrated into a “Human-Centered Quality Improvement” Approach to Solve Problems in Perinatology. Clin. Perinatol..

[13] Sarah G. Design Thinking 101. Nielsen Norman Group, 31 July 2016. www.nngroup.com/articles/design-thinking/.

[14] Charpak N., Tessier R., Ruiz J.G., Hernandez J.T., Uriza F., Villegas J., Nadeau L., Mercier C., Maheu F., Marin J. (2017). Twenty-year Follow-up of Kangaroo Mother Care Versus Traditional Care. Pediatrics.

